# Enhancing Root Growth and Water Use Efficiency of Winter Wheat by Optimizing the Irrigation Amount of Micro-Sprinkler Irrigation

**DOI:** 10.3390/plants15162540

**Published:** 2026-08-21

**Authors:** Mingda Yang, Hui Cao, Jiaju Dong, Suyu Zhang, Hongjie Liu, Jinping Chen, Xiaoyun Zheng, Shenjiao Yang, Shoutian Ma

**Affiliations:** 1 Institute of Farmland Irrigation, Chinese Academy of Agricultural Sciences, Xinxiang 453002, China; 2 Shangqiu Academy of Agricultural and Forestry Sciences, Shangqiu 476000, China; 3 College of Horticulture and Forestry, Tarim University, Alar 843300, China; 4 Key Laboratory for Crop Water Requirement and Regulation, Ministry of Agriculture and Rural Affairs of China, Xinxiang 453003, China

**Keywords:** micro-sprinkler, winter wheat, root growth, yield, water use efficiency

## Abstract

Conventional irrigation practices, characterized by excessive water application, have diminished water use efficiency (WUE) and intensified agricultural water consumption. Optimizing irrigation schedules and moderately reducing water input are therefore essential for enhancing WUE while safeguarding stable yields. Micro-sprinkler irrigation, which integrates the benefits of both drip and sprinkler systems, represents a promising technology with substantial water-saving potential. However, its effects on root development and crop yield remain inadequately investigated. Here, we established three micro-sprinkler irrigation regimes—MS20 (20 mm), MS30 (30 mm), and MS40 (40 mm)—alongside flood irrigation (FI) and rainfed (RF) controls, to systematically assess their impacts on soil water content, root growth traits, dry matter accumulation, yield, and WUE. Compared with FI, MS treatments decreased profile soil water content at jointing but increased it in specific layers during grain filling, albeit to varying extents. MS treatments enhanced total root length density (TRLD) and total root dry weight density (TRDD) relative to FI, with MS20 significantly increasing both parameters in the 20–100 cm soil layer. Moreover, MS20 increased pre-flowering dry matter translocation (PDMT) by 9.69% on average and post-flowering dry matter accumulation (PFDMA) by 15.04% during the 2022–2023 season. Yield and WUE under MS treatments exceeded those under FI by 3.46–6.53% and 4.42–21.21%, respectively. Among the MS treatments, MS20 achieved the highest average WUE. No significant yield or WUE differences were observed between MS20 and MS30, with MS30 producing the numerically maximum average yield. MS40 did not significantly affect yield in 2021–2022; however, it substantially reduced both yield and WUE relative to MS20 and MS30 in 2022–2023. A comprehensive TOPSIS analysis identified the combination of micro-sprinkler irrigation with total seasonal irrigation amounts of 80 mm or 120 mm as the optimal strategy for winter wheat production in the eastern Henan region of the North China Plain (NCP).

## 1. Introduction

The North China Plain (NCP) is one of China’s major wheat-producing areas, contributing approximately 71% of the country’s total wheat yield [1]. Nevertheless, precipitation in this region is limited and unevenly distributed, with only 100–180 mm falling during the winter wheat growing season, insufficient to meet the crop’s full developmental water demand [2]. Irrigation is therefore essential to supplement soil moisture and stabilize grain production. Although traditional surface irrigation can maintain high yields, long-term irrational irrigation methods and irrigation amounts not only reduce economic benefits but also lead to serious overdraft of groundwater resources [3], forming typical “groundwater funnel zones” [4]. Consequently, optimizing irrigation strategies is critical to reconciling high productivity with efficient water use in the NCP.

To address the severe water shortage in this region, irrigation strategies should be optimized in terms of guaranteeing water supply at critical fertility stages and reducing unproductive water loss [5]. Surface irrigation remains the predominant practice in this region, yet it exhibits low water and nutrient use efficiencies. With the advancement of water-saving agriculture and irrigation technology, more efficient alternatives such as sprinkler and drip systems are gaining wider adoption. Research indicated that drip irrigation, compared to conventional surface irrigation, can decrease water consumption by 22% and enhance water use efficiency (WUE) by 20%, all while maintaining maize yield levels [6]. Drip irrigation has consistently demonstrated significant advantages in enhancing crop yield, WUE, and nitrogen utilization across various cropping systems, outperforming both flood irrigation (FI) and rainfed conditions [7,8]. Moreover, sprinkler irrigation has proven beneficial in enhancing both yield and WUE [9,10].

Micro-sprinkler irrigation (MS) is a precision irrigation technique that integrates the benefits of both drip and sprinkler systems, offering considerable potential for enhancing water conservation and crop productivity [11]. Studies have shown that, compared to FI, MS can improve WUE by 19.35% and reduce evapotranspiration by 14.20%. Under the same irrigation volume, yield and WUE can be increased by 4.6% and 11.7%, respectively [12]. Under this irrigation method, an irrigation amount of 120 mm has been found to optimize both yield and WUE [11]. However, although optimal irrigation regimes have been identified, the underlying mechanisms contributing to high yield and efficiency under micro-sprinkler irrigation remain underexplored. Further research is needed to determine whether its potential for stable yield and water savings can be further enhanced.

As the main organs responsible for absorbing water and nutrients, roots play a vital role in supporting aboveground biomass growth, yield production, and improving the efficiency of resource utilization [5]. An ideal root spatial distribution, particularly in deeper soil layers, reflects a crop’s enhanced capacity for accessing subsoil water and nutrients [13,14], and constructing ideal root architecture has become a key target for crop breeding to improve water and nutrient utilization [15]. Previous studies have demonstrated that moderate water regulation can stimulate deeper root growth, thereby facilitating greater access to deep soil moisture and improving WUE [13]. Enhancing water use efficiency is also a core goal of crop precision improvement, which can be achieved through regulating plant structure and water utilization processes [16]. As a deep-rooted crop, winter wheat typically concentrates the majority of its roots within the 0–40 cm soil layer, though roots may extend to depths of up to 2 m, enabling utilization of deep water resources [9]. Micro-sprinkler irrigation has been reported to increase root length density (RLD) below the 80 cm depth, contributing to improved yield and WUE [1].

Moreover, applying a moderate level of rehydration after drought stress during the jointing stage has been shown to increase root length density (RLD) in the 0–20 cm soil layer and promote a more favorable root distribution throughout the 0–100 cm profile [5]. These results highlight the potential of regulating the irrigation amount and utilizing precision irrigation strategies to facilitate deeper root water uptake, thereby enhancing WUE and crop productivity.

Currently, research on micro-sprinkler irrigation mainly considers crop yield and resource utilization efficiency through crop growth, crop nitrogen distribution, and water consumption patterns [11,17,18], while less research has been conducted on efficient crop water utilization and yield enhancement mechanisms from the perspective of root growth and root distribution. Moreover, crop responses to management vary across ecological zones and climatic contexts, leading to divergent optimal practices. Consequently, micro-sprinkler irrigation scheduling must be tailored to local conditions rather than generalized. Based on these considerations, we hypothesized that optimizing irrigation methods and applying moderate water restrictions would enhance root development and distribution in deeper soil layers, promote post-anthesis dry matter accumulation and grain filling, and ultimately improve both yield and WUE.

To address these issues, a two-year field experiment was carried out, incorporating three irrigation modes—micro-sprinkler irrigation, conventional surface (flood) irrigation, and rainfed treatment—alongside varying irrigation quotas within the micro-sprinkler system. The study aimed to (1) investigate how micro-sprinkler irrigation influences root development and spatial distribution; (2) assess the contributions of dry matter accumulation and translocation to yield formation and water use efficiency; and (3) determine the most suitable micro-sprinkler irrigation strategy for winter wheat cultivation in the eastern Henan region of the North China Plain (NCP).

## 2. Materials and Methods

### 2.1. Experiment Site

**Figure 1:**
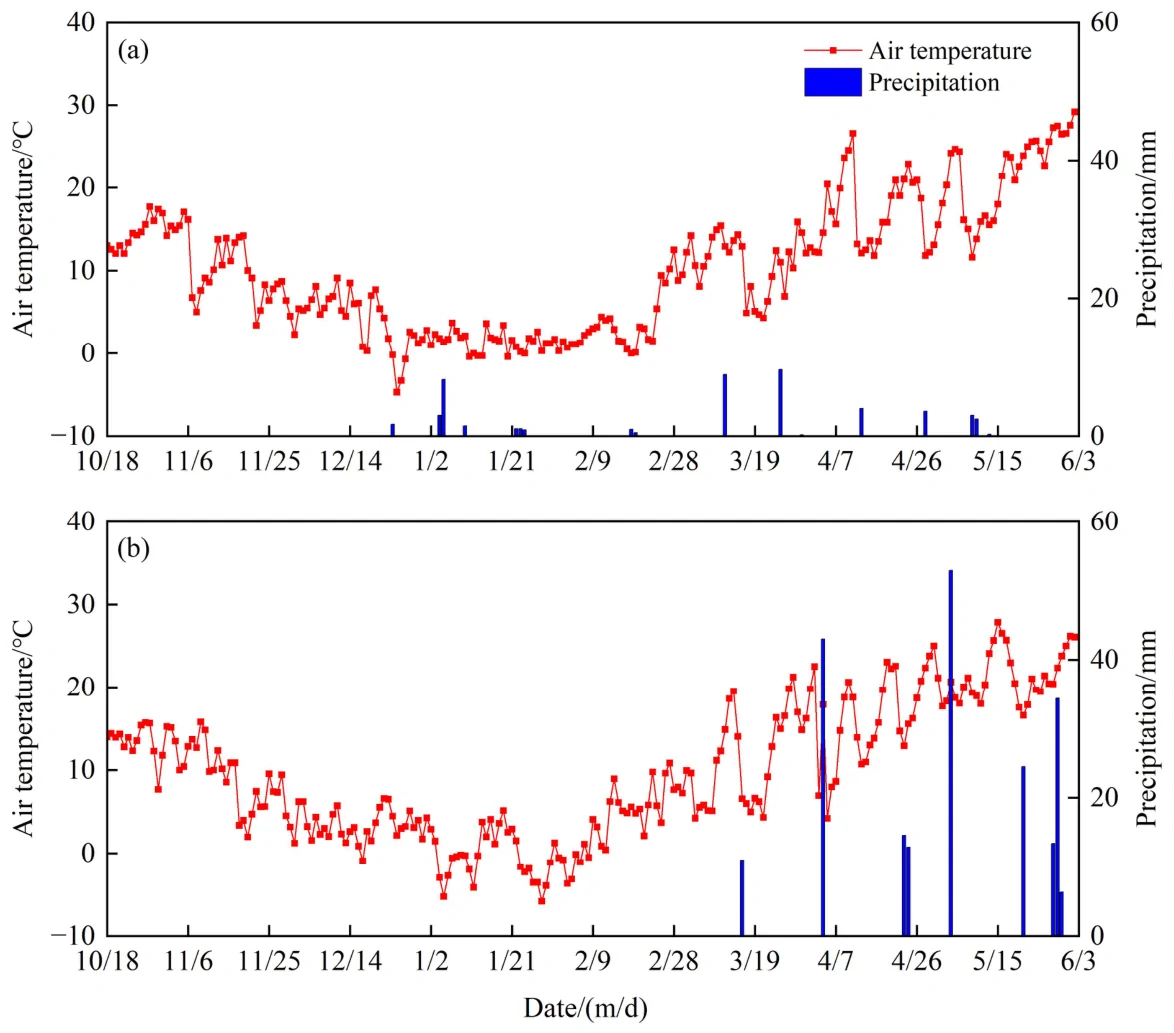
The daily average temperature and precipitation during the growth seasons of 2021–2022 (**a**) and 2022–2023 (**b**).

A field experiment was carried out from 2021 to 2023 at the National Field Scientific Observation Station for Agricultural Ecosystems in Shangqiu, Henan Province, China (34°34′ N, 115°33′ E; altitude 55.6 m). The area experiences an average annual temperature of 13.9 °C, with long-term mean annual evapotranspiration and precipitation recorded at 1735 mm and 708 mm, respectively. Notably, only 232.8 mm of precipitation occurs during the winter wheat season, representing 32.9% of the annual total—insufficient to meet the crop’s full water requirements. Therefore, supplemental irrigation is essential to sustain optimal plant development. The experimental soil is classified as tidal soil, with bulk density of 1.39 g cm^−3^ in the top 0–30 cm and a field capacity of 25.52 g g^−1^. Baseline soil fertility data are detailed in Table 1, whereas the meteorological conditions during the winter wheat growing season are illustrated in Figure 1.

### 2.2. Experimental Design

Five irrigation treatments were set up: a rainfed (RF), conventional flood irrigation (FI), and three micro-sprinkler irrigation regimes with varying water quotas—MS20 (20 mm), MS30 (30 mm), and MS40 (40 mm). Each treatment was replicated three times, resulting in 15 plots, each with an area of 90 m^2^. To prevent lateral water movement and maintain plot independence, a 1.5 m buffer zone separated adjacent plots. The RF treatment received no irrigation throughout the growing season. In the FI treatment, irrigation was applied twice—70 mm each at the jointing and flowering stages (total irrigation amount of 140 mm). For the MS treatments, irrigation was applied four times: at the jointing, heading, flowering, and grain filling stages. The total irrigation quotas for MS20, MS30, and MS40 were 80 mm, 120 mm, and 160 mm, respectively. Micro-sprinkler irrigation was delivered using a standard 7-hole belt with a spray radius ranging from 1.0 to 1.2 m. To ensure uniform water distribution, three micro-sprinkler belts were installed parallel to one another within each plot.

The winter wheat cultivar Shangmai 167 (*Triticum aestivum* L.) was selected for this experiment, which is a widely planted crop in the eastern Henan Province. It was a self-developed variety by the Shangqiu Academy of Agricultural and Forestry Sciences. Wheat was sown on 7 November 2021 and 18 October 2022, with 20 cm row spacing. To ensure uniform seedling emergence, seeding rates were set at 300 kg ha^−1^ in 2021 due to the delayed planting date, and 187.5 kg ha^−1^ in 2022. Before sowing, a basal application of compound fertilizer (N:P_2_O_5_:K_2_O = 20:17:17) was applied at 600 kg ha^−1^. For the micro-sprinkler irrigation (MS) treatments, nitrogen was topdressed at 30 kg ha^−1^ during each of the jointing, flowering, and grain filling stages. In comparison, the rainfed (RF) and flood irrigation (FI) treatments received a single nitrogen dose of 90 kg ha^−1^ at the jointing stage via manual broadcast. The specific irrigation and quantity of the topdressing nitrogen fertilizer applied for each treatment are shown in Table 2. All other field operations conformed to standard local agronomic practices.

### 2.3. Indicators and Methods

#### 2.3.1. Soil Water Content (SWC)

During the critical reproductive stage of wheat, soil samples were collected from the 0–100 cm soil profile at 20 cm intervals using a soil auger. The samples were then oven-dried at 105 °C to a constant weight to determine the soil water content (SWC).

#### 2.3.2. Root Length Density and Root Dry Matter Density

At the late filling stage of winter wheat (25 May 2022 and 23 May 2023), root sampling was conducted at a representative location within each plot. Root sampling was conducted in both the intra-row and inter-row areas using a root auger with a 7.0 cm inner diameter, at 20 cm depth intervals across the 0–100 cm soil profile. Samples collected from identical depths were subsequently pooled into composite samples. Following a 24 h water soak, root samples were placed in a 15 mm mesh sieve and gently rinsed to separate them from soil, with impurities and roots from other plants removed using tweezers. 

Root morphological characteristics were analyzed by scanning the samples with an Epson V850 Pro scanner (Seiko Epson Corporation, Suwa-shi, Nagano Prefecture, Japan), followed by image processing using the WinRHIZO Pro Vision 2009c software package (Regent Instruments Inc., Québec City, QC, Canada). After scanning, the root samples were oven-dried at 80 °C until a constant weight was achieved. The dry mass of roots was then measured using a high-precision analytical balance with an accuracy of 0.0001 g. According to the method described by Jha et al. [19], roots were stratified into three depth intervals: 0–20 cm, 20–60 cm, and 60–100 cm. Root length density (RLD) and root dry weight density (RDD) were subsequently calculated using standard formulas:

(1) $$RLD=\frac{L}{V}$$

(2) $$RDD=\frac{D}{V}$$

where RLD is the density of root length (cm cm^−3^); RDD is the density of root dry matter; L is root length (cm); D is root dry matter (mg); and V is the volume of the soil sample (cm^3^).

#### 2.3.3. Dry Matter Accumulation and Distribution

At both the flowering and maturity stages, plants in two 30 cm-long rows were sampled from each plot. The harvested plant material was first dried at 105 °C for 30 min, followed by further drying at 75 °C until a constant weight was reached. Based on the method proposed by Barman et al. [20], calculations were performed for dry matter (DM) accumulation, translocation, and distribution:

(3) PDMT=PDMA - MDMA

(4) $$PDMTR=\frac{\mathrm{PDMT}}{\mathrm{FDMA}}\;\times \;100\%$$

(5) $$CPDMT=\frac{\mathrm{PDMT}}{\mathrm{MYMA}}\;\times \;100\%$$

PFDMA = MYMA − PDMT (6)

(7) $$CPFDMA=\frac{\mathrm{PFDMA}}{\mathrm{MYMA}}\;\times \;100\%$$

where PDMT is the pre-flowering dry matter translocation amount (kg ha^−1^); PDMA is pre-flowering dry matter accumulation (kg ha^−1^); MDMA is dry matter accumulation in nutrient organs at maturity (kg ha^−1^); PDMTR is the pre-flowering dry matter translocation rate (%); FDMA is the dry matter accumulation of nutrient organs during flowering (kg ha^−1^); CPDMT is the contribution of pre-flowering dry matter translocation to grain (%); MYMA is grain dry matter accumulation at maturity (kg ha^−1^); PFDMA is post-flowering dry matter accumulation (kg ha^−1^); and CPFDMA is the contribution of post-flowering dry matter accumulation to grain (%).

#### 2.3.4. Yield and Yield Components

Winter wheat was harvested from an undisturbed 1 m^2^ area (1 m × 1 m) within each plot after physiological maturity. The number of spikes was recorded, and grain yield was determined following threshing, with grain moisture content adjusted to a standard of 13%. Additionally, thirty representative wheat plants located adjacent to the sampling area were selected for the measurement of kernel number per spike and 1000-grain weight (TGW).

#### 2.3.5. Evapotranspiration

Winter wheat evapotranspiration was determined by a soil water balance equation [21]:

ET = I + P + SWD − D − R + CR (8)

where ET is evapotranspiration (mm); I is the irrigation amount with the stage of wheat growth (mm); P is precipitation (mm); SWD is the change in water storage from 0–100 cm soil layers (mm); D is downward drainage below the 100 cm soil layer (mm); *R* is surface runoff (mm); and CR is capillary rise into the 100 cm soil layer (mm). In our study, D, R, and CR were all zero.

#### 2.3.6. Harvest Index, Water Use Efficiency, and Irrigation Water Use Efficiency

Indicators were calculated according to the following equations:

(9) $$HI=\frac{Y}{AB}$$

(10) $$WUE=\frac{Y}{ET}$$

(11) $$IWUE=\frac{Y_{I}-Y_{0}}{I}$$

where HI is harvest index (%); WUE is water use efficiency (kg ha^−1^ mm^−1^); IWUE is irrigation water use efficiency (kg ha^−1^ mm^−1^); Y is grain yield (kg ha^−1^); Y_I_ is grain yield with irrigation treatment (kg ha^−1^); Y_0_ is grain yield with no irrigation treatment (kg ha^−1^); ET is evapotranspiration (mm); and I is irrigation amount (mm).

### 2.4. Comprehensive Evaluation

In order to comprehensively evaluate the effects of treatments on the growth, development, and resource utilization efficiency of winter wheat, a multi-indicator comprehensive evaluation and analysis was carried out by using the TOPSIS method based on the indicators of root characteristic parameters, dry matter accumulation and transfer, aboveground biomass, ET, WUE, HI, and yield as well as its component factors.

### 2.5. Data Analysis

Excel 2010 was used for data sorting and management, while Origin 2025 facilitated graphical visualization and Pearson correlation analysis. Statistical differences were examined through analysis of variance (ANOVA assumptions of homogeneity) using SPSS 24.0, with significance levels determined by the LSD test at *p* < 0.05. To evaluate the importance of each variable in influencing yield, a random forest regression model was applied.

## 3. Results and Analysis

### 3.1. Changes in Soil Water Content (SWC) at Different Growth Stages

**Figure 2:**
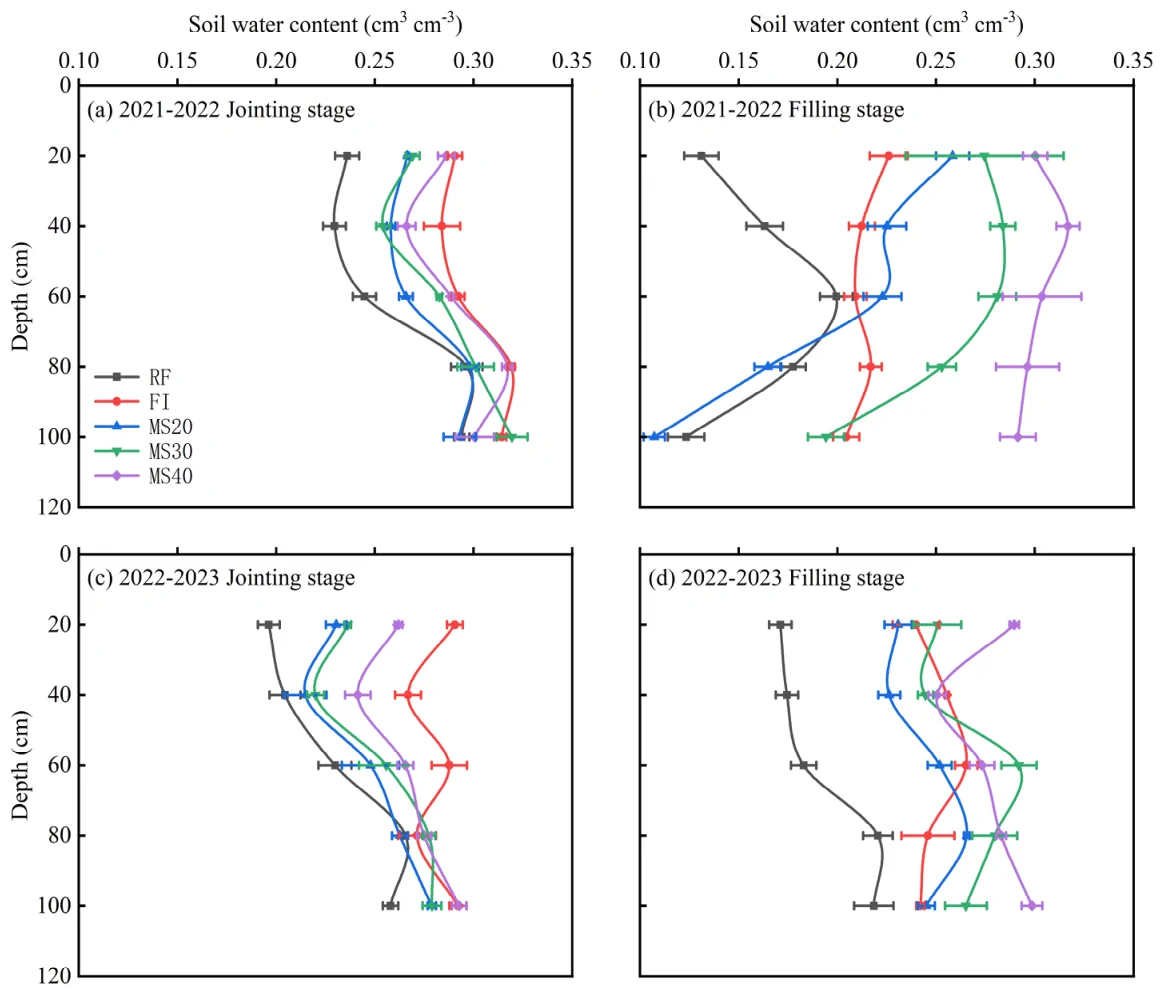
The effects of different irrigation methods and micro-sprinkling amounts on soil water content during the winter wheat growing seasons of 2021–2022 and 2022–2023. RF: rainfed; FI: wild flooding irrigation; MS20: micro-sprinkling 20; MS30: micro-sprinkling 30; and MS40: micro-sprinkling 40.

Soil water content (SWC) demonstrated marked fluctuations across different growth stages under varying irrigation strategies and micro-sprinkler quotas (Figure 2). At the jointing stage, pronounced differences in SWC within the 0–60 cm soil layer were recorded, with the FI treatment showing the highest moisture levels, while the lowest were consistently detected in the RF plots. During the grain filling phase, SWC under RF remained persistently low, whereas other treatments displayed interannual variability. In the 2021–2022 season, relative to FI, SWC within the top 60 cm of soil increased by 9.08%, 29.63%, and 42.18% under MS20, MS30, and MS40, respectively. Conversely, in the 60–100 cm layer, a notable reduction of 35.45% in SWC was observed only under MS20. For the 2022–2023 season, SWC in the 0–60 cm zone increased by 3.52% and 6.95% in the MS30 and MS40 treatments, respectively, while a decrease of 6.79% was recorded under MS20. In contrast, deeper soil layers (60–100 cm) exhibited enhanced SWC, with increases of 4.62%, 11.66%, and 19.19% corresponding to MS20, MS30, and MS40, respectively.

### 3.2. Root Characteristics

#### 3.2.1. Root Length Density

**Figure 3:**
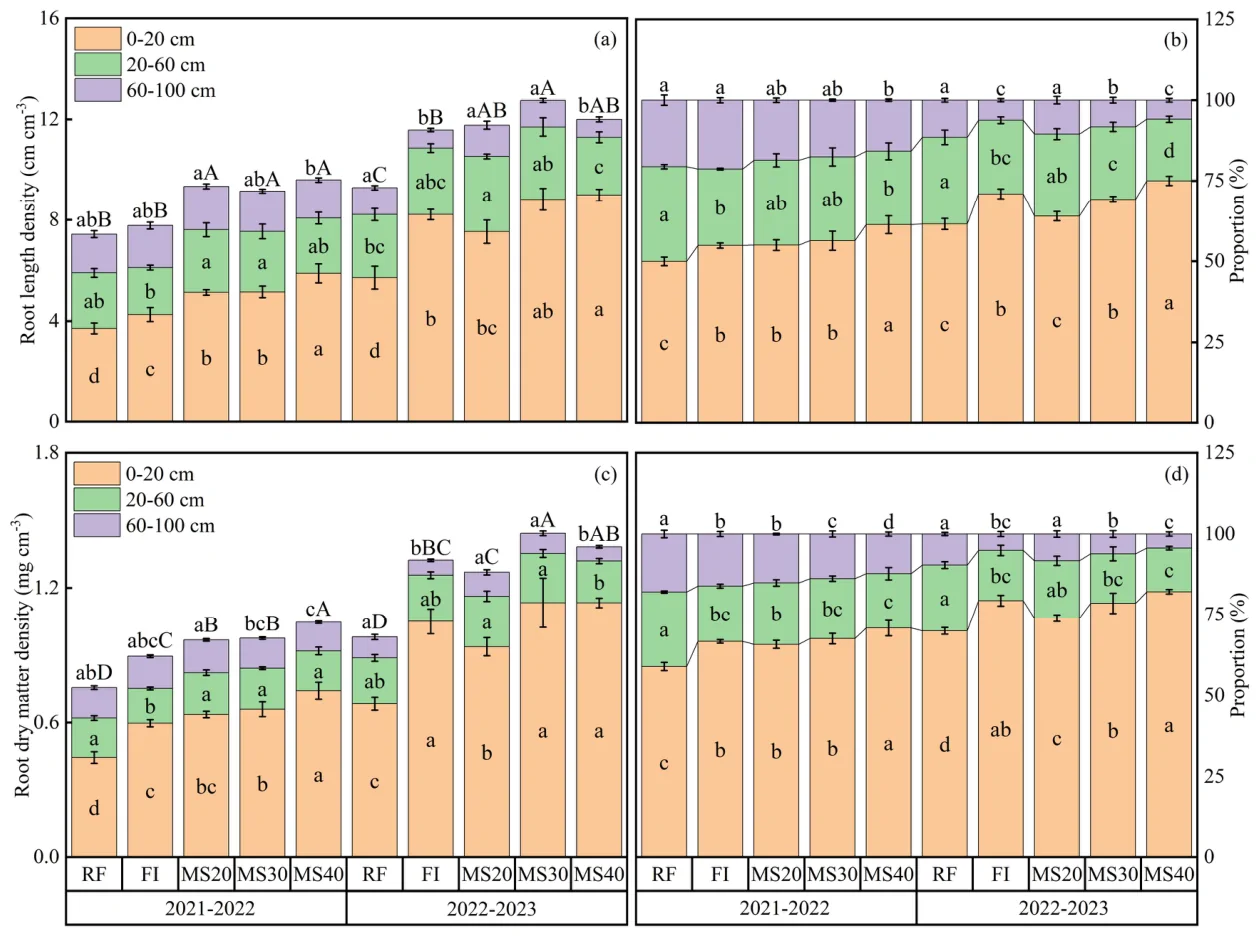
The effects of different irrigation methods and micro-sprinkling amounts on root length density (**a**), percentage of root length density in different soil layers to total root length density (**b**), root dry matter density (**c**), and percentage of root dry matter density in different soil layers to total dry matter density (**d**). RF: rainfed; FI: wild flooding irrigation; MS20: micro-sprinkling 20; MS30: micro-sprinkling 30; and MS40: micro-sprinkling 40. Different lowercase letters indicate significant differences in root length density, root dry weight density from different soil layers and its proportion among different treatments. Different uppercase letters indicate significant differences in root length density and root dry weight density from the whole soil profile (0–100 cm) among different treatments.

Over the full soil profile (0–100 cm), micro-sprinkler irrigation resulted in a 23.15–37.39% increase in total root length density (TRLD) compared with the rainfed (RF) treatment, and a 3.63–23.42% increase relative to flood irrigation (FI). Nevertheless, significant differences among treatments were only detected in the 2021–2022 growing season (Figure 3a, *p* < 0.05). The total root length density (TRLD) under the MS30 and MS40 treatments was 12.03% and 19.25% higher, respectively, than that under flood irrigation (FI), and exceeded that under the rainfed (RF) treatment by 48.30% and 57.86%, respectively, in the 0–20 cm soil layer. In deeper layers (20–100 cm), MS20 exhibited the greatest RLD. Specifically, within the 20–60 cm depth, MS20 increased RLD by 16.63% and 21.56% compared to RF and FI, respectively. In the 60–100 cm layer, the increases reached 14.62% and 25.21%, respectively.

As illustrated in Figure 3b, the 0–20 cm soil layer was found to contain the highest proportion of RLD (PRLD) under all treatments. Within this layer, the MS40 treatment exhibited the highest PRLD, exceeding that of the RF and FI treatments by 22.25% and 8.36%, respectively. In contrast, in the 20–100 cm profile, higher PRLD values were observed under the RF and MS20 treatments. Specifically, PRLD in the 20–60 cm layer increased by 20.80% and 11.22% under RF and MS20 compared to FI, respectively, while in the 60–100 cm layer the respective increases were 16.59% and 5.77%.

#### 3.2.2. Root Dry Matter Density

As illustrated in Figure 3c, total root dry weight density (TRDD) varied significantly among treatments throughout the soil profile (*p* < 0.05). Relative to the FI treatment, MS30 and MS40 treatments increased RDD by 9.05% and 9.54%, respectively, whereas MS20 treatment exhibited a 4.03% reduction in RDD between 2022 and 2023. In the upper 0–20 cm layer, RDD exhibited a similar pattern to that observed in the entire profile, with MS30 and MS40 treatments seeing significant increases of 8.82% and 13.80% compared to FI (*p* < 0.05), respectively. Deeper in the profile (20–100 cm), MS20 had the greatest RDD. Specifically, in the 20–60 cm segment, MS20 enhanced RDD by 8.81% and 14.14% over RF and FI, respectively, while in the 60–100 cm layer the increases reached 10.66% and 20.32%.

According to the vertical distribution of root dry weight density (RDD), the proportion of RDD (PRDD) in the 0–20 cm layer was greater under MS40 and FI treatments, exceeding that of MS20 by 9.48% and 4.54%, respectively (Figure 3d). Conversely, the RF treatment exhibited the lowest PRDD in this surface layer. Within the 20–60 cm depth, PRDD values under RF and MS20 treatments were relatively higher, surpassing those of FI by 32.99% and 12.65%, respectively. In the 60–100 cm layer, during the 2022–2023 season, the PRDD in MS20 and MS30 increased by 62.94% and 20.47%, respectively, compared to FI treatment.

### 3.3. Dry Matter Accumulation and Translocation

There were significant differences in pre-flowering dry matter translocation (PDMT), translocation efficiency (PDMTR), and contribution to grain yield (CPDMT) among treatments (Table 3, *p <* 0.05). PDMT was highest in the MS20 and MS30 treatments, which were 9.69% and 7.23% higher than the FI treatments, respectively; PDMTR was higher in the RF and MS20 treatments, which were 19.19% and 13.73% higher than the FI treatments, respectively. Under micro-sprinkler irrigation, PDMTR was 7.13% and 14.36% higher in the MS20 treatment than the MS30 and MS40 treatments, respectively; CPDMT differed in years, but was generally highest in the RF treatment.

The highest post-flowering dry matter accumulation (PFDMA) was recorded under the MS treatments. Compared to the FI treatment, increases of 15.04% and 17.17% in PFDMA were observed in the MS20 and MS30 treatments, respectively, during the 2022–2023 growing season. Interannual variation was noted in the contribution of post-flowering dry matter accumulation to grain (CPFDMA), with the lowest values consistently observed in the RF treatment. In 2021–2022, only the MS40 treatment had 2.04% higher CPFDMA compared to the FI treatment, while in 2022–2023 the MS20 and MS30 treatments had 1.57% and 4.42% higher CPFDMA, respectively.

### 3.4. Aboveground Biomass and Grain Yield

Aboveground biomass under micro-sprinkler irrigation showed significant difference in 2021–2022 (Table 4, *p <* 0.05). In the two-year study, only the aboveground biomass of the MS40 treatment increased by 1.56% (2021–2022) and 6.05% (2022–2023) compared to the FI treatment. Significantly fewer number of spikes (*p <* 0.05) were produced under the RF treatment compared to both the FI and MS treatments, whereas no significant difference was detected between the FI and MS treatments. For kernel number per spike (KNS), no significant variation was detected among the MS treatments, though MS20 and MS30 showed increases of 5.31% and 8.11%, respectively, compared to the FI treatment. There were interannual differences in 1000-grain weight (TGW), with the mean value of TGW in 2022–2023 being 15.76% lower than that in 2021–2022, with the RF and MS20 treatments being less affected (by 12.82% and 14.35%). TGW of the MS20, MS30, and MS40 treatments was increased by 4.07%, 7.21%, and 3.74%, respectively, compared to the FI treatment. Meanwhile, the yield under micro-irrigation conditions was significantly different from the FI treatment only in 2022–2023 (*p <* 0.05). Compared to the FI treatment, average yields under the MS20, MS30, and MS40 treatments increased by 6.53%, 8.13%, and 3.46%, respectively, with the yield of MS30 being only 1.52% higher than that of the MS20 treatment. The harvest index was higher in the MS20 and MS30 treatments, which were 7.08% and 3.88% higher than the FI treatment, respectively.

### 3.5. ET, WUE, and IWUE

ET under the FI and MS40 treatments was significantly higher than that of the other treatments (Table 5, *p <* 0.05). The average ET was 12.26% and 9.26% lower in the MS20 and MS30 treatments, respectively, compared to the FI treatment. In terms of WUE, the FI treatment showed significantly lower values than all other treatments. Compared to the FI treatment, the WUE of the MS20, MS30, and MS40 treatments could be increased by 21.21%, 19.37%, and 4.42%, respectively. Meanwhile, among the MS treatments, the average WUE of the MS20 and MS30 treatments was 16.13% and 14.42% higher than that of the MS40 treatment, respectively. IWUE was highest under the MS20 treatment, with a 146.72% increase compared to the FI treatment.

### 3.6. Correlation Between Indicators

**Figure 4:**
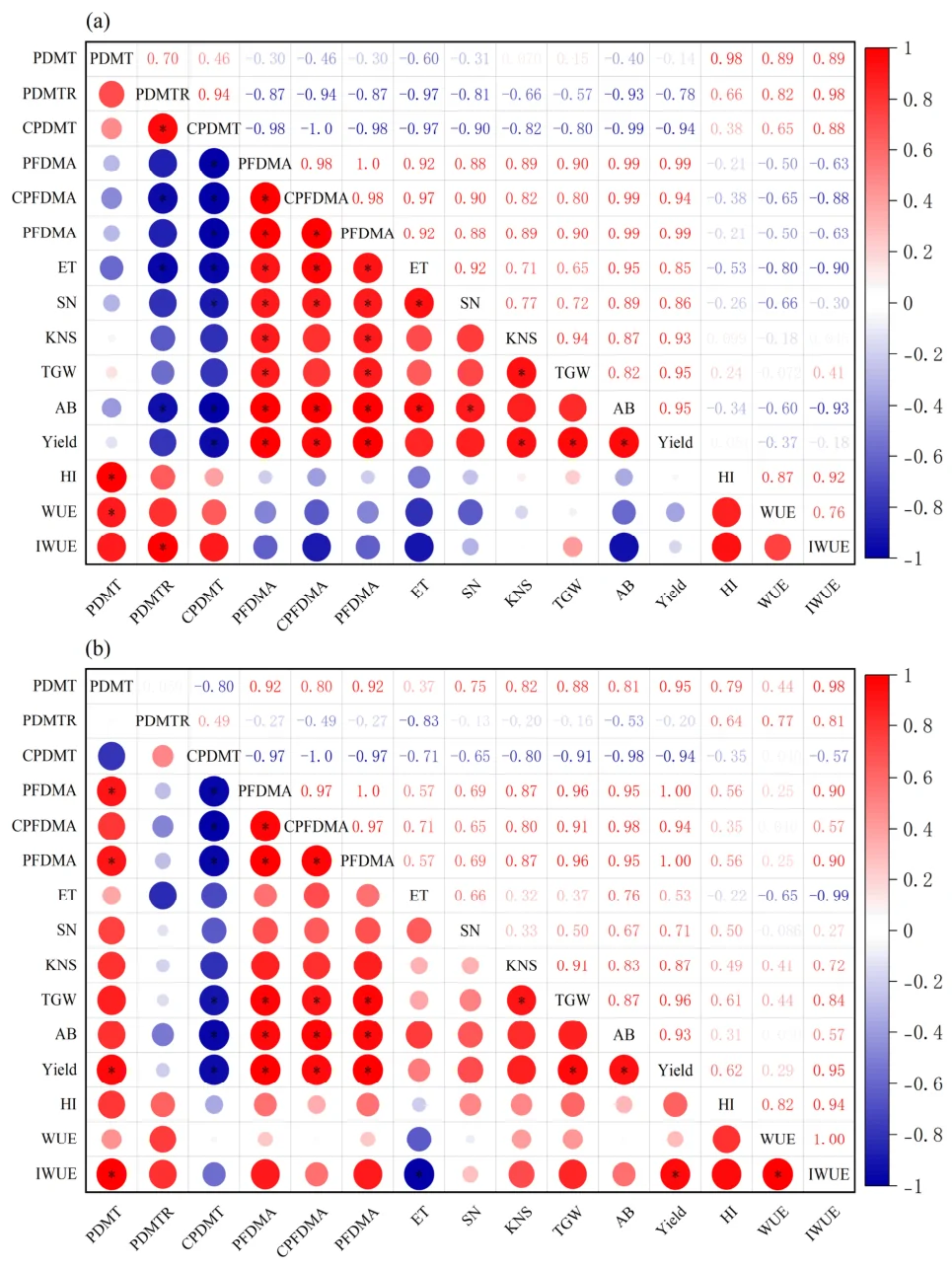
Correlation among indicators under (**a**) 2021–2022 and (**b**) 2022–2023. TRLD, the density of total root length; TRDD, total root dry weight density; PDMT, pre-flowering dry matter translocation; PDMTR, pre-flowering dry matter translocation rate; CPDMT, contribution of pre-flowering dry matter translocation to grain; PFDMA, post-flowering dry matter accumulation; CPFDMA, contribution of post-flowering dry matter accumulation to grain; AB, aboveground biomass; ET, evapotranspiration; SN, spike number; KNS, kernel number per spike; TGW, 1000-grain weight; Yield: grain yield; HI, harvest index; WUE, water use efficiency; and IWUE, irrigation water use efficiency. * indicate statistical significance at probability values of 0.05.

**Figure 5:**
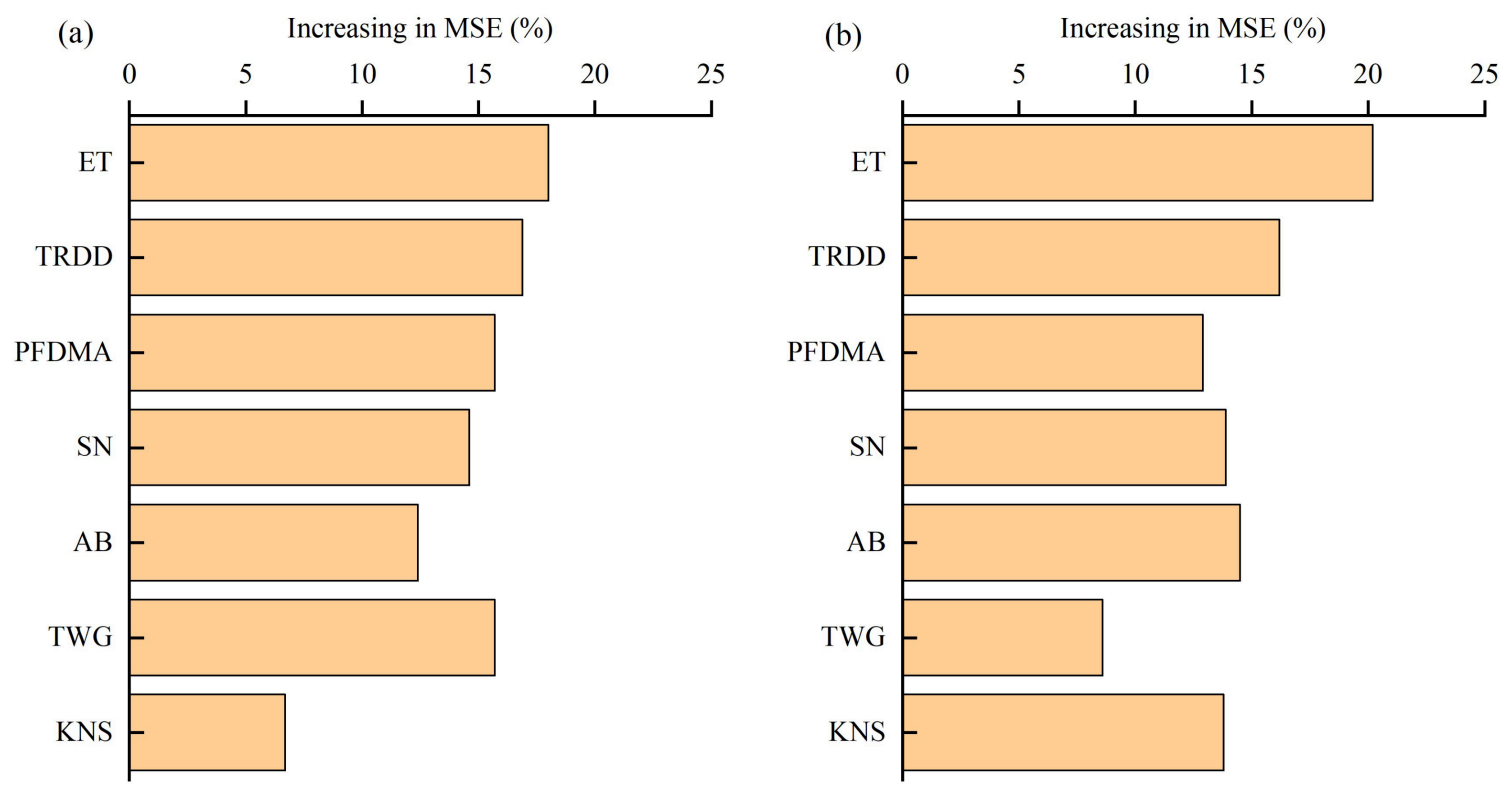
The random forest mean predictor importance of the stock of yield and other indicators under (**a**) 2021–2022 and (**b**) 2022–2023. MSE: Mean Squared Error; ET, evapotranspiration; TRDD, total root dry weight density; PFDMA, post-flowering dry matter accumulation; AB, aboveground biomass; SN, the number of spikes; KNS, kernel number per spike; and TGW, 1000-grain weight.

Winter wheat root characterization parameters were correlated with aboveground parameters (Figure 4). Yield was significantly correlated (*p <* 0.05) with root characterization parameters, PFDMA, aboveground biomass, TGW, and kernel number per spike (KNS), and the correlation coefficients of TRDD and PFDMA were 0.93 (2021–2022) and 0.82 (2022–2023), respectively, and the PFDMA and aboveground biomass correlation coefficients were 0.99 (2021–2022) and 0.95 (2022–2023), respectively. The random forest regression analysis identified evapotranspiration (ET) and total root dry weight density (TRDD) as the primary determinants of yield (Figure 5). The influence of individual yield components varied between years: in 2021–2022, TGW and spike number (SN) were the dominant contributors to yield, whereas in 2022–2023, SN and KNS played a more prominent role.

Although random forest analysis identified TRDD as the most important predictor of yield across treatments, it is noteworthy that the MS20 treatment did not always exhibit the highest TRDD (Figure 3c). This apparent discrepancy implies that yield is not simply determined by the absolute magnitude of root biomass, but rather by the functional efficiency of roots—particularly their spatial distribution and activity during the post-anthesis period. In this context, the MS20 treatment promoted a higher proportion of roots in deeper soil layers (20–100 cm), which likely enhanced water and nitrogen uptake during grain filling, when surface soil moisture is often depleted. Thus, TRDD as a whole-profile indicator may reflect overall root system size, but its predictive power for yield arises from its association with deep-root functionality, rather than total root mass per se. This interpretation is supported by the positive correlation between yield and deep-layer RLD/RDD (Figure 4), and the higher PFDMA observed under MS20 (Table 3).

### 3.7. Comprehensive Evaluation of Irrigation Management for Winter Wheat

The comprehensive effects of irrigation method and water quota on TRLD, TRDD, PDMT, PDMTR, CPDMT, PFDMA, CPFDMA, aboveground biomass, ET, SN, KNS, TGW, yield, HI, and WUE were evaluated using the TOPSIS method (Table 6). The results indicated that the RF treatment consistently received the lowest ranking, followed by the FI treatment. In contrast, the MS20 and MS30 treatments achieved the highest overall performance across both growing seasons of winter wheat. These findings highlight that advanced irrigation strategies, when paired with optimal irrigation amounts, can effectively enhance both yield and WUE in a coordinated manner.

## 4. Discussion

### 4.1. Effects of Different Irrigation Methods and Micro-Sprinkler Irrigation Amounts on Soil Water Content, Root Characteristics

Root growth and distribution patterns largely determine the water and nutrient uptake capacity of crops, which in turn affects yield formation and resource utilization efficiency [22]. An increase in root length density (RLD) means an increase in the number of roots per unit of soil volume, which enhances the crop’s ability to obtain soil water and nutrients [23]. Soil moisture status directly affects root distribution across soil depths [24]. It has been shown that optimizing irrigation methods and irrigation regimes is beneficial to improve the distribution of roots in different soil layers and enhance their expansion to deeper soils, thus improving crop adaptation to drought stress [19]. Mulched drip irrigation was able to increase soil water content and its distribution uniformity and optimize specific root length and root–shoot ratio, which in turn suppressed aboveground over-nutritional growth compared to traditional flood irrigation [25], and consequently reduced transpiration. Meanwhile, Li et al. [1] showed that micro-irrigation treatments (drip and micro-sprinkler) increased RLD in the 80–200 cm soil layer compared to traditional flood irrigation treatments, which in turn improved water uptake and utilization by roots. In this study, the FI treatments had higher soil water content than the MS and RF treatments at the jointing stage and lower soil water content than the MS treatments (MS30 and MS40) at the filling stage (Figure 2). This was attributed to the fact that the filling stage is a critical fertility period, which requires more water for proper crop growth and grain filling [26]. Moreover, RLD and RDD were higher in the MS treatments than in the FI treatments (except for RDD in the MS20 treatment in 2022–2023) (Figure 3). This may be attributed to the MS treatment improving soil porosity and reducing evapotranspiration compared to the FI treatment [11,27], which provided a more favorable growth environment for the root, thus promoting root growth. It was worth noting that RLD and RDD showed a decreasing trend in MS treatments after the irrigation quota exceeded 30 mm (MS30) (Figure 3). This was attributed to the fact that water deficit leads to root stunting, whereas more water inhibits root respiration and increases nutrient leaching [14,28]. Furthermore, roots tend to take up water preferentially from upper soil layers when soil water availability in this zone is favorable [29,30]. Compared with the MS20 treatment, higher soil water content under the MS30 and MS40 treatments restricted root penetration into deeper soil layers, as favorable soil moisture in upper layers could satisfy the water demand for plants.

Furthermore, the distribution of roots across soil layers plays distinct roles in water uptake. One study has shown that a higher root distribution in the top soil layer plays a decisive role in crop water uptake [10], while when the crop is in the reproductive growth stage large amounts of photosynthetically active products will be transported to the grains, which reduces the utilization of photosynthetic carbon sources by the root, inhibits root growth, and accelerates the senescence and death of the root [25,31], which can negatively affect yield and WUE. Compared with shallow roots, the quality of deep roots during the post-flowering period was more critical for water uptake and grain filling in wheat [32]. This further confirms that optimizing root vertical distribution conforms to the design concept of ideal crop root architecture [15]. Irrigation methods also influence root vertical distribution patterns. Flood irrigation increased root distribution in the surface soil and inhibited root growth in the deeper soil compared to drip irrigation [33]. Meanwhile, Uddin et al. [34] reported that the water utilization as well as growth of dryland wheat are highly correlated with root length in deep soil. In the present study, the MS20 and FI treatments exhibited no significant differences in PRLD and PRDD within the upper 0–20 cm soil layer. However, significantly higher PRLD and PRDD values were observed under MS20 in the deeper layers of 20–60 cm and 60–100 cm (Figure 3b,d). This distribution pattern indicates that MS20 effectively inhibits root proliferation in the surface soil while promoting root growth in deeper layers, thereby enhancing the crop’s capacity to access and utilize water from deeper soil profiles more efficiently.

The random forest results (Figure 5) further highlight that TRDD, rather than aboveground traits alone, plays a dominant role in determining yield. However, the lack of a one-to-one correspondence between TRDD and yield ranking across treatments suggests that root quality (e.g., deep-root proportion, physiological activity) outweighs root quantity in driving yield formation under micro-sprinkler irrigation. This is consistent with the TOPSIS evaluation (Table 6), where MS20 ranked highest due to its balanced performance in root distribution, dry matter translocation, and WUE, despite not having the maximum TRDD.

### 4.2. Effects of Different Irrigation Methods and Micro-Sprinkler Irrigation Amounts on Dry Matter Distribution, Yield, and WUE

Dry matter accumulation and harvest index (HI) are important bases for achieving high yields. This study demonstrated that the biomass under MS treatments was comparable to, or in some cases exceeded, that observed under FI treatment, accompanied by a general upward trend in yield (Table 4). Interestingly, the biomass of the FI treatment was higher than that of the MS20 treatment in 2021–2022, but the harvest index was instead lower, which may be related to the synergy between reproductive and nutrient organs [35]. Pre-flowering dry matter accumulation was usually closely related to the photosynthetic capacity of crop leaves [36], which implied that carbohydrates accumulated by the nutrient organs before flowering provide sufficient initial energy for post-flowering grain filling [37]. In this study, the MS20 and MS30 treatments significantly improved pre-flowering dry matter translocation and its efficiency compared to the FI treatment, indicating that appropriate irrigation and moderate water deficit could help improve root distribution and the soil environment, which in turn could enhance nutrient uptake and light utilization efficiency, as well as promote coordinated aboveground growth.

Moreover, grain yield is directly influenced by the accumulation of photosynthetic assimilates produced after flowering [38]. It has been reported that post-flowering dry matter accumulation (PFDMA) plays a decisive role in yield formation, while variations in irrigation regimes or soil moisture status regulate photosynthetic efficiency. When the crop is subjected to water stress, the photosynthetic rate decreases, leading to abnormal grain growth and shortened time of the seed filling rate, and decreased yield [39]. Micro-sprinkler irrigation can effectively reduce evapotranspiration, increase chlorophyll content and net photosynthetic rate compared to flood irrigation [12,40], and thus increase crop yield. This study showed that the PFDMA of the MS treatments was comparable to that of the FI treatment in 2021–2022, while both PFDMA and CPFDMA were higher in the MS treatments than in the FI treatment in 2022–2023 (Table 3). MS treatments could sustain or boost PFDMA, laying a material foundation for the improvement of final grain yield. For the MS treatments, increasing the irrigation amount elevated PFDMA in 2021–2022, whereas in 2022–2023 the treatment with a high irrigation amount (MS40) significantly reduced PFDMA. This may be due to the large differences in meteorological conditions [41]. The precipitation during the heading to filling stage was 9.4 mm (4 times) and 159 mm (8 times) in 2021–2022 and 2022–2023, respectively. Moderate precipitation can improve the microclimate in farmland, mitigate heat stress, enhance canopy photosynthesis, and thereby promote post-anthesis dry matter accumulation [42,43]. In contrast, excessive rainfall during critical phenological stages—particularly grain filling—can elevate disease occurrence [44,45], hasten or prematurely terminate grain filling, and consequently diminish dry matter accumulation and depress yield performance. In addition to biomass accumulation and translocation, final crop yield is largely determined by its yield components. Gaining insight into how these components respond to different irrigation methods and water volumes is essential for elucidating yield formation mechanisms and optimizing irrigation strategies.

Yield components, such as spike number (SN), kernel number per spike (KNS), and 1000-grain weight (TGW), determined the increase in crop yield. Currently, increasing KNS and TGW is an important means of increasing yield. It has been shown that irrigation increases yield by increasing SN and KNS [46]. Yield reduction was strongly associated with the number of spikes per unit area when soil water deficit was moderate, while yield reduction was strongly associated with KNS when soil water deficit was severe [47]. Meanwhile, irrigation methods also had important effects on yield and constitutive factors. Alternate surface and subsurface irrigation (ADI) reduced SN but increased yield by increasing grain weight compared to surface drip irrigation (DI) [48]. Relative to traditional flood irrigation, micro-sprinkler irrigation has been shown to enhance grain yield through the improved partition of post-flowering dry matter and optimization of yield components [10]. However, the results of Liu et al. [49] showed that micro-sprinkler irrigation increased KNS and TGW while decreasing SN compared to conventional flood irrigation, but ultimately increased the yield as well. Thus, there is variability in the results shown at different locations even for the same type of irrigation. In this study, yield was significantly correlated with KNS and TGW (Figure 4), and the MS20 treatment as well as MS30 treatments were able to increase KNS and TGW (Table 3). This indicates that suitable soil moisture conditions are favorable to the crop’s reuse of light resources and promote grain formation.

Water use efficiency (WUE) is regarded as a crucial indicator for evaluating sustainable agricultural practices. Improving WUE is not only related to irrigation regulation but also closely associated with crop physiological and structural optimization [16]. Previous studies have reported that, under micro-sprinkler irrigation (MS), WUE surpasses that of conventional flood irrigation (FI) when the irrigation volume exceeds 60 mm, with WUE initially increasing and then decreasing as irrigation amount rises—peaking at a threshold of 120 mm [50]. However, the present study revealed a consistently higher WUE under MS across all treatments relative to FI, though a decreasing trend was observed with increasing irrigation amount (Table 4). Notably, the MS20 treatment (80 mm) yielded the maximum WUE, indicating a lower optimal threshold than previously reported. This variation could be explained by the imbalance between water input and the corresponding yield increase [47]. In this study, deep drainage was neglected during the calculation of ET, especially for the FI treatment. This assumption may be reasonable in 2021–2022, since scarce rainfall in March (jointing stage) and April (flowering stage) resulted in low soil water content. In 2022–2023, the irrigation applied to the FI treatment at the flowering stage might generate a small amount of deep drainage, owing to the cumulative rainfall of 71.1 mm in April (flowering stage). This would likely overestimate ET and consequently underestimate the WUE of the FI treatment. Nevertheless, we consider such effects to be limited, and this discrepancy will not alter the quantitative relationship between the FI treatment and the MS20 as well as MS30 treatments, as the WUE of the MS20 and MS30 treatments was substantially higher relative to the FI treatment (Table 5). For follow-up research, deep drainage could be quantified using traditional means (such as deeper soil moisture measurement) or novel technical approaches (such as numerical simulation) [51], which would improve the accuracy, reliability, and reference value of our results.

In this study, compared with the FI treatment, the frequent low-volume irrigation pattern of MS treatments optimized the soil water distribution in the soil profile during the filling stage, and promoted the growth of deep roots and plants. Soil water movement is the main driving force of nutrient migration [52]; therefore, the spatial distribution of soil nitrogen is tightly coupled with soil moisture distribution. MS treatments can prevent nitrate nitrogen from migrating into deeper soil owing to its frequent low-volume irrigation amount. Meanwhile, MS treatments increased the proportion of root distribution in deep soil layers, which could promote the root uptake of nitrate nitrogen from deep soil, reducing the accumulation of nitrate nitrogen in subsoil [1]. All these processes are beneficial to improving the plant uptake and utilization of soil nitrate nitrogen. Furthermore, micro-sprinkling irrigation allows split and delayed nitrogen fertilizer application, which increases the concentration of nitrate nitrogen in the root zone and sustains a sufficient nutrient supply in the late growing period, and helped guarantee post-anthesis photosynthetic production [11,52]. Significant interactive effects between water and nitrogen were observed under fertigation [53]. Water–nitrogen synergy may improve plant and root growth, strengthen the absorption and utilization of soil water and nitrogen, facilitate grain filling, and further elevate grain yield and WUE under micro-sprinkling irrigation. Therefore, the superior grain yield and WUE achieved under micro-sprinkler irrigation resulted from the interactive effects of water and nitrogen, rather than a single factor.

Meanwhile, in the present study, TOPSIS was applied to comprehensively assess plant development, yield performance, and WUE, thereby determining the most suitable irrigation method and water quota (Table 6). Nevertheless, the investigation did not extend to evaluating the capacity of deep roots for water absorption, which limits a full understanding of their role in improving WUE. To address this gap, future studies may consider employing stable isotope techniques involving hydrogen and oxygen to quantitatively trace water uptake by deep roots, offering critical insights into optimizing resource use efficiency.

## 5. Conclusions

Compared to the FI treatment, MS treatments with small and frequent water applications not only reduced ET but also optimized soil moisture conditions during the filling stage, increased TRLD and TRDD, enhanced PDMT, and maintained a high level of PFDMA. Ultimately, these treatments improved yield, WUE, and IUE. MS20 treatment can optimize root distribution in the soil profile, improve the percentage of TRLD and TRDD in the deeper soil layer (20–100 cm), and increase PDMT and PDMTR in both years as well as PFDMA in the second year, and ultimately lead to higher yields compared to the FI treatment. The MS20 treatment significantly increased WUE because it had a lower ET compared to the FI treatment. With the increase in micro-sprinkling irrigation amount, the yield of winter wheat did not increase continuously. Moreover, the PFDMA, TGW, and yield of the MS40 treatment were significantly lower than those of the MS20 and MS30 treatments under the condition of heavy rainfall during the filling stage.

(1) MS treatment increased TRLD and TRDD in the full soil profile compared to the FI treatment, and the MS20 treatment increased the percentage of TRLD and TRDD in the deeper soil layer (20–100 cm).
(2) The PDMT and PDMTR of the MS20 treatment increased by 9.69% and 13.73%, respectively, compared to the FI treatment, and the PFDMA of the MS20 treatment increased by 15.04% compared to the FI treatment in 2022–2023.
(3) The yield and WUE of the MS treatments were 3.46–6.53% and 4.42–21.21% higher than the FI treatment, respectively, with the MS20 treatment performing best.
(4) The TOPSIS model indicated that micro-sprinkler irrigation combined with 80 mm or 120 mm irrigation was favorable for coordinating root growth, dry matter accumulation, yield, and WUE, and was a suitable irrigation system for winter wheat growth.

## Figures and Tables

**Table 1:** The base nutrient data for the 0–30 cm soil layer.

Total Nitrogen	Organic Matter	Available Nitrogen	Available Phosphorus	Available Potassium
g kg^−1^	g kg^−1^	g kg^−1^	g kg^−1^	g kg^−1^
0.9	14.55	87.11	16.45	137.35

**Table 2:** The treatments and irrigation amounts used in the present study.

Treatments	Irrigation (mm)					Topdressing Nitrogen Fertilizer (kg ha^−1^)			
	Jointing Stage	Heading Stage	Flowering Stage	Filling Stage	Total	Jointing Stage	Flowering Stage	Filling Stage	Total
RF					0	90			
FI	70		70		140	90			
MS20	20	20	20	20	80	30	30	30	90
MS30	30	30	30	30	120	30	30	30	90
MS40	40	40	40	40	160	30	30	30	90

**Table 3:** Dry matter accumulation and translocation characteristics of winter wheat under different irrigation methods and micro-sprinkling amounts.

Year	Treatments	Dry Matter Before Anthesis			Dry Matter After Anthesis	
		Translocation Amount (kg ha^−1^)	Translocation Rate (%)	Contribution Rate to Grain (%)	Accumulation Amount (kg ha^−1^)	Contribution Rate to Grain (%)
2021–2022	RF	3437.12 ab	30.60 a	45.21 a	4167.21 c	54.81 c
	FI	3227.24 b	23.42 c	35.13 bc	5974.34 ab	64.92 ab
	MS20	3508.04 a	27.84 b	38.14 b	5710.54 b	61.91 b
	MS30	3544.36 a	26.62 b	36.94 bc	6049.45 ab	63.14 ab
	MS40	3272.41 b	23.92 c	33.72 c	6427.35 a	66.33 a
2022–2023	RF	3269.01 c	26.82 a	44.10 a	4143.22 c	55.90 c
	FI	3484.23 bc	24.71 ab	40.11 b	5196.23 b	59.91 b
	MS20	3853.05 a	27.03 a	39.20 bc	5976.15 a	60.84 ab
	MS30	3652.11 ab	24.42 ab	37.51 c	6091.13 a	62.52 a
	MS40	3526.21 bc	23.92 b	40.16 b	5276.25 b	59.90 b

Note: lowercase letters indicate significant differences among different treatments (*p <* 0.05).

**Table 4:** Yield and aboveground biomass of winter wheat under different irrigation methods and micro-sprinkling amounts.

Years	Treatment	Spike Number	Kernel Number Per Spike	1000-Grain Weight	Yield	Aboveground Biomass	HI
		(×10^4^ ha^−1^)		(g)	(kg ha^−1^)	(kg ha^−1^)	(%)
2021–2022	RF	455 b	35.30 c	45.82 d	7604.11 b	15,416.00 d	0.49 a
	FI	545 a	37.60 ab	48.39 c	9201.05 a	19,790.00 ab	0.47 a
	MS20	535 a	37.40 ab	50.17 b	9218.53 a	18,350.00 c	0.50 a
	MS30	518 a	38.80 a	52.22 a	9593.23 a	19,351.67 b	0.50 a
	MS40	526 a	37.70 ab	51.57 ab	9699.42 a	20,098.67 a	0.48 a
2022–2023	RF	548 b	33.70 b	39.95 c	7412.05 c	16,351.67 c	0.45 a
	FI	610 a	33.90 b	41.11 b	8680.12 b	18,981.33 b	0.46 a
	MS20	614 a	37.90 a	42.98 a	9829.05 a	20,272.33 a	0.49 a
	MS30	582 ab	38.50 a	43.74 a	9743.23 a	21,047.33 a	0.46 a
	MS40	585 ab	36.90 a	41.29 b	8802.42 b	20,130.00 a	0.44 a

Note: lowercase letters indicate significant differences among different treatments (*p <* 0.05).

**Table 5:** Water use efficiency and irrigation water use efficiency of winter wheat under different irrigation methods and micro-sprinkling amounts.

Years	Treatments	I	ET	WUE	IWUE
		(mm)	(mm)	(kg ha^−1^ mm^−1^)	(kg ha^−1^ mm^−1^)
2021–2022	RF	--	239.82 c	31.72 a	--
	FI	140	344.64 a	26.72 b	11.40 c
	MS20	80	304.52 b	30.28 a	20.23 a
	MS30	120	301.43 b	31.86 a	16.61 ab
	MS40	160	337.31 a	28.75 ab	13.14 bc
2022–2023	RF	--	260.14 d	28.56 a	--
	FI	140	365.05 a	23.80 b	9.10 c
	MS20	80	318.13 c	30.94 a	28.42 a
	MS30	120	342.53 b	28.45 a	20.45 b
	MS40	160	367.04 a	23.99 b	9.82 c

Note: I represents irrigation amount; ET represents evapotranspiration; WUE represents water use efficiency; and IWUE represents irrigation water use efficiency. Lowercase letters indicate significant differences among different treatments (*p <* 0.05).

**Table 6:** TOPSIS-based comprehensive evaluation.

Treatments	2021–2022				2022–2023			
	D+	D−	C	Rank	D+	D−	C	Rank
RF	3.03	2.22	0.42	5	3.25	1.86	0.36	5
FI	2.71	2.18	0.45	4	2.44	1.97	0.45	4
MS20	1.35	2.77	0.67	2	1.10	3.24	0.75	1
MS30	1.24	3.1	0.71	1	1.72	3.07	0.64	2
MS40	2.06	2.89	0.58	3	2.43	2.08	0.46	3

Note: D+ and D− represent positive ideal solution distance and negative ideal solution distance, respectively; C represents the composite score index. The larger the value, the higher the score.

## Data Availability

The datasets used or analyzed during the current study are available from the corresponding author on reasonable request.
